# Somatic mutation dynamics in MDS patients treated with azacitidine indicate clonal selection in patients-responders

**DOI:** 10.18632/oncotarget.22957

**Published:** 2017-12-06

**Authors:** Kamila Polgarova, Karina Vargova, Vojtech Kulvait, Nina Dusilkova, Lubomir Minarik, Zuzana Zemanova, Michal Pesta, Anna Jonasova, Tomas Stopka

**Affiliations:** ^1^ Department Biocev, First Faculty of Medicine, Charles University, Vestec, Czech Republic; ^2^ Department of Haematology, First Faculty of Medicine and General Hospital, Charles University, Prague, Czech Republic; ^3^ Department of Pathophysiology, First Faculty of Medicine, Charles University, Prague, Czech Republic; ^4^ Department of Cytogenetics, First Faculty of Medicine and General Hospital, Charles University, Prague, Czech Republic; ^5^ Faculty of Mathematics and Physics, Charles University, Prague, Czech Republic

**Keywords:** somatic mutation, MDS, AML, azacitidine, NGS

## Abstract

Azacitidine (AZA) for higher risk MDS patients is a standard therapy with limited durability. To monitor mutation dynamics during AZA therapy we utilized massive parallel sequencing of 54 genes previously associated with MDS/AML pathogenesis. Serial sampling before and during AZA therapy of 38 patients (reaching median overall survival 24 months (Mo) with 60% clinical responses) identified 116 somatic pathogenic variants with allele frequency (VAF) exceeding 5%. High accuracy of data was achieved via duplicate libraries from myeloid cells and T-cell controls. We observed that nearly half of the variants were stable while other variants were highly dynamic. Patients with marked decrease of allelic burden upon AZA therapy achieved clinical responses. In contrast, early-progressing patients on AZA displayed minimal changes of the mutation pattern. We modeled the VAF dynamics on AZA and utilized a joint model for the overall survival and response duration. While the presence of certain variants associated with clinical outcomes, such as the mutations of *CDKN2A* were adverse predictors while *KDM6A* mutations yield lower risk of dying, the data also indicate that allelic burden volatility represents additional important prognostic variable. In addition, preceding 5q- syndrome represents strong positive predictor of longer overall survival and response duration in high risk MDS patients treated with AZA. In conclusion, variants dynamics detected via serial sampling represents another parameter to consider when evaluating AZA efficacy and predicting outcome.

## INTRODUCTION

Upon ageing the somatic mutations, via yet unknown process, accumulate in genes encoding various epigenetic regulators that under normal conditions regulate transcription. These genes include DNA methylase *DNMT3A*, Polycomb Group Protein *ASXL1*, and Methylcytosine Dioxygenase *TET2* [1]. Individuals bearing the ageing-associated variants may develop Myelodysplastic syndromes (MDS) with red cell, platelet, and myeloid cytopenias and differentiation blockades. MDS often progress to acute myeloid leukemia (AML) - a condition marked by myeloblast accumulation. Genetic aberrations in MDS or AML occur in a stem cell that gains survival properties and outcompeting advantage marked by proliferation of myeloid and granulocytic-monocytic progenitor pools [2] often without altering lymphopoiesis. Strikingly, inhibiting the DNA methylation (DNMTi) in MDS either with 5-Azacitidine (AZA) [3] or 5-aza-2′deoxycytidine (Decitabine) [4] can induce complete remission (CR) or at least improve hematologic parameters (HI) in up to two thirds of MDS patients. Use of DNMTi including AZA therapy has superior response rate compared to conventional chemotherapy (84% vs. 37%) with median duration ∼20 months [5]. Progression of MDS on AZA leads to MDS/AML that has very limited therapeutic options. It represents yet largely unrecognized process involving resistance of stem/progenitor populations to AZA in the MDS patient bone marrow [6].

Previous studies suggested that altered function of the ageing-associated epigenetic modulators was involved in resistance to DNMTi. A study of 213 MDS patients of all IPSS risk categories displayed approximately fifty percent response to DNMTi and the significant association of response with *TET2* mutation. However, co-occurrence of *TET2* and *ASXL1* or *ASXL1* mutation alone was more prevalent in a non-response group [7] supporting the possibility that a combination of mutations determines the DNMTi therapy outcome. Interestingly, mutations in *TP53* gene associated with shorter overall survival but not with response [7]. Another study containing 107 MDS patients treated by DNMTi concluded that none of the 26 analyzed MDS genes associated with the response to DNMTi, however, the *TP53* and *DNMT3A* mutations again occurred in patients with shorter overall survival and AML-free survival [8]. Another study involving 134 AZA-treated patients of all IPSS risk categories confirmed a negative impact of *TP53* variants on overall survival, however, none of the mutated genes associated with the response rate [9]. Unexpectedly, variants in histone modifiers genes (*EZH2*, *ASXL1*) in univariate analysis associated with prolonged survival on AZA [9]. Recently, the study involving 41 MDS patients that were sequenced before and after the progression to AML [10] indicated that mutations in genes encoding *ASXL1*, *RUNX1*, and *TP53* were often present in post-progression samples albeit the statistical power of these data was quite limited in such type of study.

Here we addressed the genetics of AZA treatment using serial sampling and strengthen the accuracy of mutation detection by introducing duplicate library sequencing of myeloid population and non-tumorous T-cell control samples to call somatic variants specifically. Our data extend the previous work that suggested existence of substantial somatic variant dynamics during AZA therapy further supporting the notion of relationship of such dynamics and clinical outcome.

## RESULTS

### Mutation dynamics in the AZA-treated MDS patients

**Figure 1:**
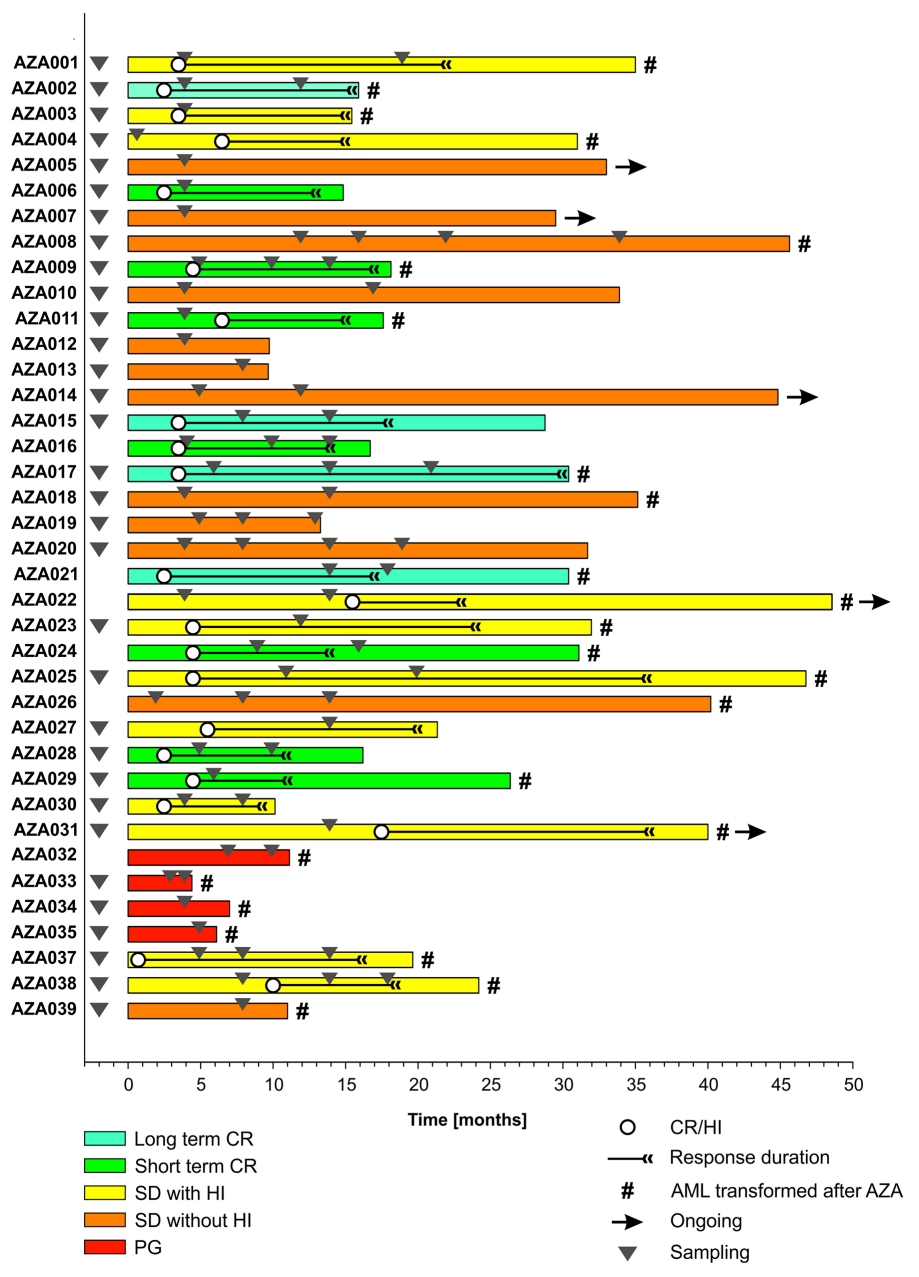
Swimmers’ plot of 38 patients treated with AZA Responses (in different colors: CR, SD, HI, or PG), their duration (in months), sampling (triangles), and AML transformation (^#^) are indicated.

**Figure 2:**
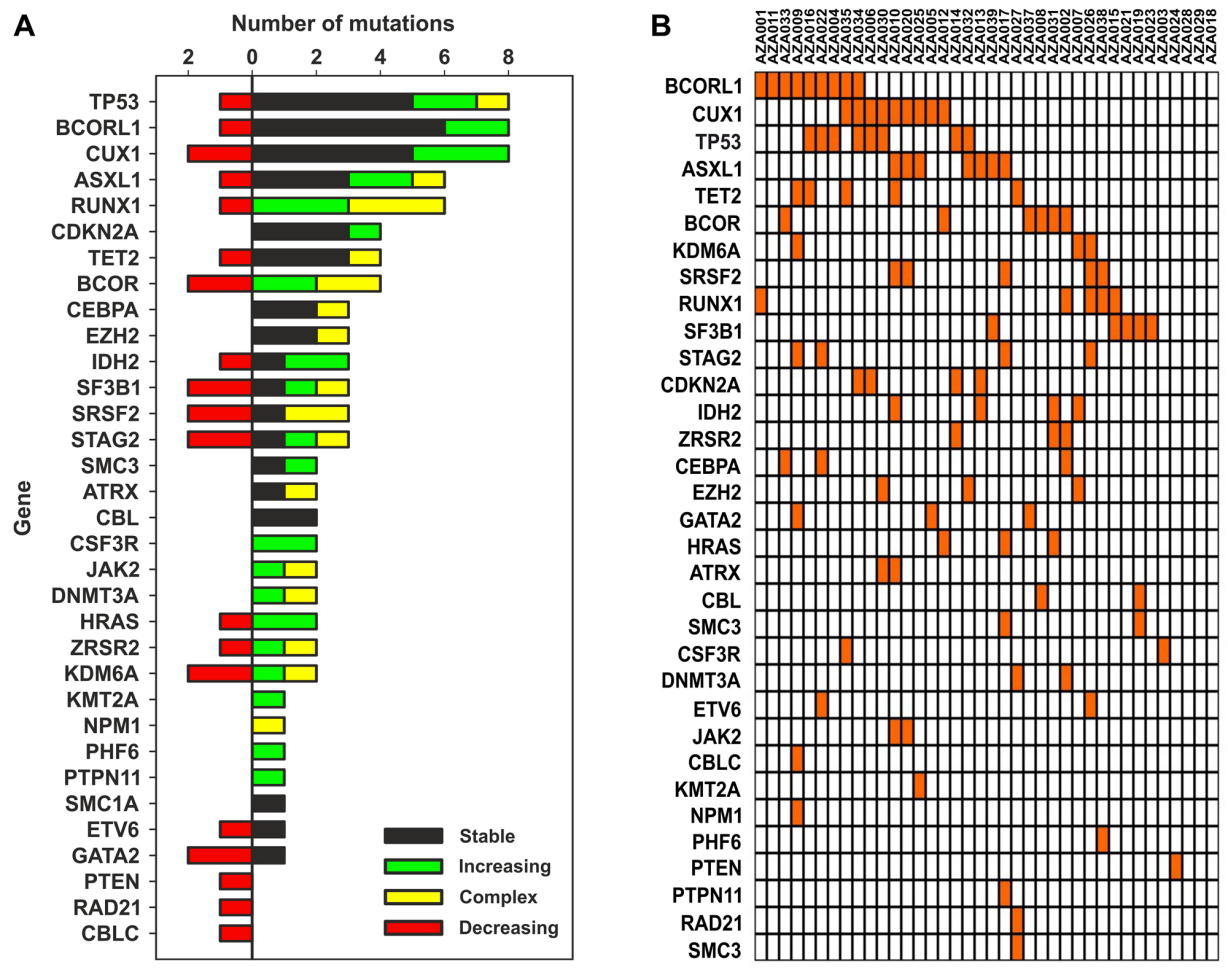
**(A)** Number of MDS variants (horizontal axis) in genes listed on the vertical axis. Colors indicate dynamics of the variants. Variants are also listed within the Supplementary Table 2, the data were obtained by NGS utilizing the TruSight kit (see M&M). **(B)** Mutation heatmap (row: genes, column: patients), unmutated genes are not listed.

To identify variants either tolerated or repelled with the AZA therapy we tracked BM samples during AZA treatment by the next generation sequencing (NGS). Clinical data of studied patients, such as the follow up duration, response, sampling, and progression are shown in the Figure 1. Patient cohort data are summarized in the Supplementary Table 1. The NGS platform was the TruSight Myeloid Sequencing Panel (Illumina, San Diego, USA) that is a set of 568 amplicons and 108 kilobases designed to detect somatic variants of 54 genes previously associated with myeloid malignancies. 92% of our MDS cohort bore at least one somatic mutation with mostly 3 mutations per patient (range 1-8). Sum of the detected 116 somatic variants with their particular dynamic pattern (in colors) is shown in the Figure 2A. When comparing mutations during AZA therapy we noted that approximately half of the mutations were quite stable and did not vary in their VAF more than two fold while other variants displayed increasing or decreasing trends of their allelic burden following the AZA therapy. Next we constructed a heat map documenting all somatically mutated genes per patient that excluded polymorphisms and variants bellow 5% of allelic burden (Figure 2B). The most abundant were variants in *CUX1* (N=10, 8.6%), *TP53* (N=9, 7.8%), *BCORL1* (N=9, 7.8%), *ASXL1* (N=7, 6%), *RUNX1* (N=7, 6%), *BCOR* (N=6, 5.2%), *TET2* (N=5, 4.3%), *SF3B1* (N=5, 4.3%), *SRSF2* (N=5, 4.3%), *STAG2* (N=5, 4.3%), *IDH2* (N=4, 3.4%) and *CDKN2A* (N=4, 3.4%). 28 out of 116 mutations were insertions or deletions (in/dels); the majority were single nucleotide variants (N=82) or complex mutations (N=6). Expectedly, 43% of the detected variants matched to the COSMIC database of recurrently mutated genes in cancer implicating that these gene variants may be more frequently called in randomly sequenced MDS samples. Indeed, thirteen of these 116 variants recurred also in our MDS cohort. Supplementary Table 3 summarizes all detected variants and VAFs at each time point together with clinical, laboratory and cytogenetic data.

### Mutation dynamics relate to the MDS clinical course

**Figure 3:**
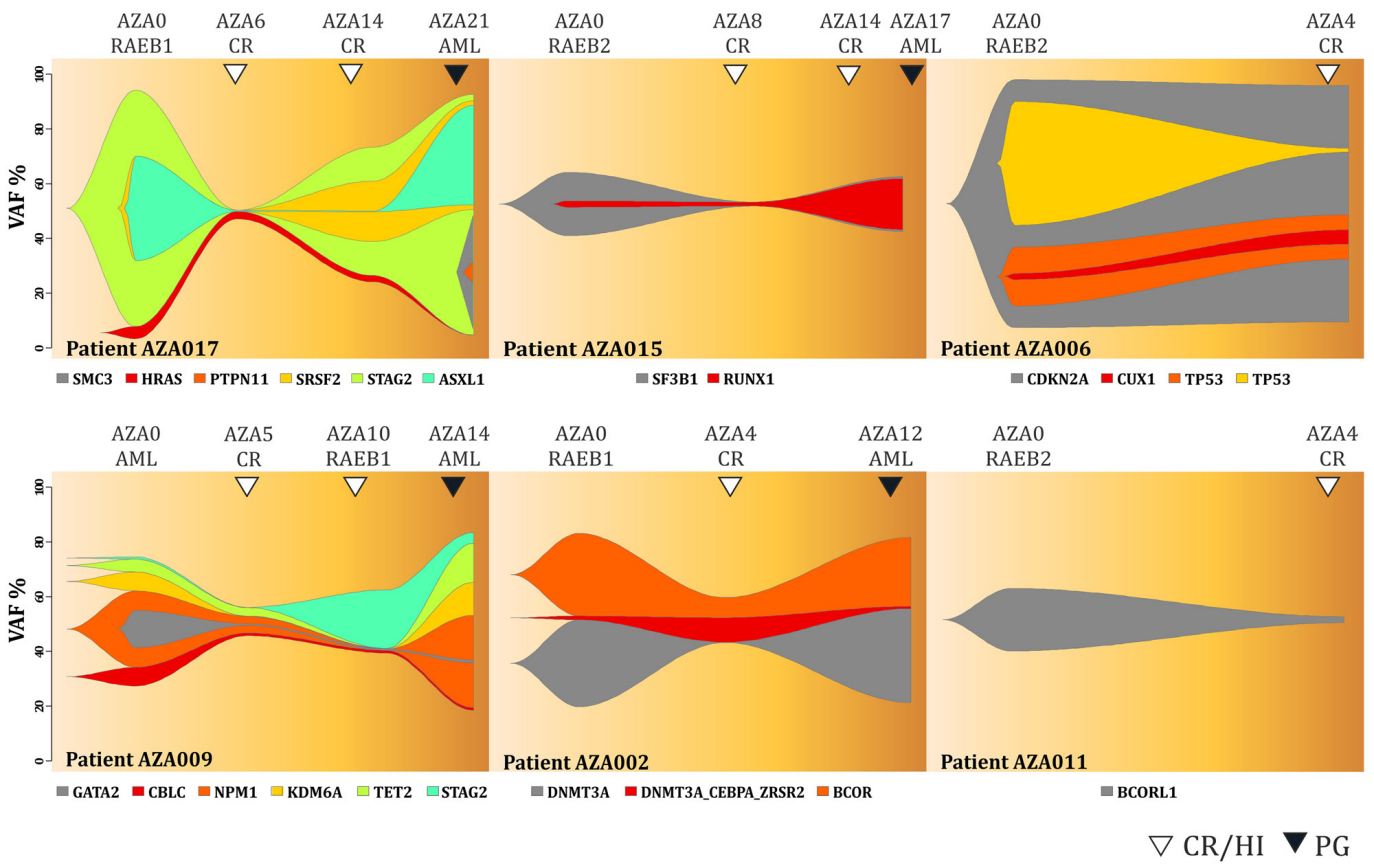
Fish plots of somatic variants detected in MDS patients achieving complete remission Arrows indicate CR (empty) and transformation to AML (PG, dark). Y-axis represents VAF (%) and X-axis is the time in weeks.

As pointed above, we noted substantial mutation dynamics of the variants in patients before and during AZA therapy. Figure 3 shows data of 6 (out of 8) patients that achieved complete remission (CR) according to the IWG criteria visualized by the fish plots that are time course plots modeling most likely changes in the clonal architecture of somatic mutations. We observed that the AZA therapy led to the marked decrease of the mutations’ allelic burden at the CR time point. Over time, these mutations at later time points reappeared upon AZA (thus preceding the clinical progression) indicating that these variants could not be controlled by AZA. For example, patient AZA017 (upper left) displayed mutations in *STAG2* (VAF=0.95), *ASXL1* (0.43), *SRSF2* (0.42) and *HRAS* (0.07) at diagnosis while after 6 cycles of AZA these mutations were barely detectable; all had VAF<0.03. Interestingly, while still in remission, at the bone marrow restaging after 14 cycles of AZA, the previously detected mutations of *STAG2*, and *SRSF2* had markedly reappeared and this was followed later by *ASXL1*. Finally, at progression stage after 21 cycles of AZA all mutations from diagnosis were restored (to almost original VAF) and the two new mutations developed in *PTPN11* (VAF=0.09) and *SMC3* (VAF=0.45). We noted that AZA017 was the only patient that on top of the reappearance of previously detected mutations had developed additional mutations upon AML progression. Pattern of the mutation reappearance was observed also in the patient AZA009. In additional 4 patients (AZA002, AZA006, AZA011, AZA015) the variants’ dynamics involved less complex changes but always included a loss of the mutation pattern upon achievement of the CR but at the same time these patients never reached a mutation-free status. This supports the clinical view that MDS patients receiving CR upon AZA may relapse with the clone/s bearing initial variants identified prior AZA therapy.

**Figure 4:**
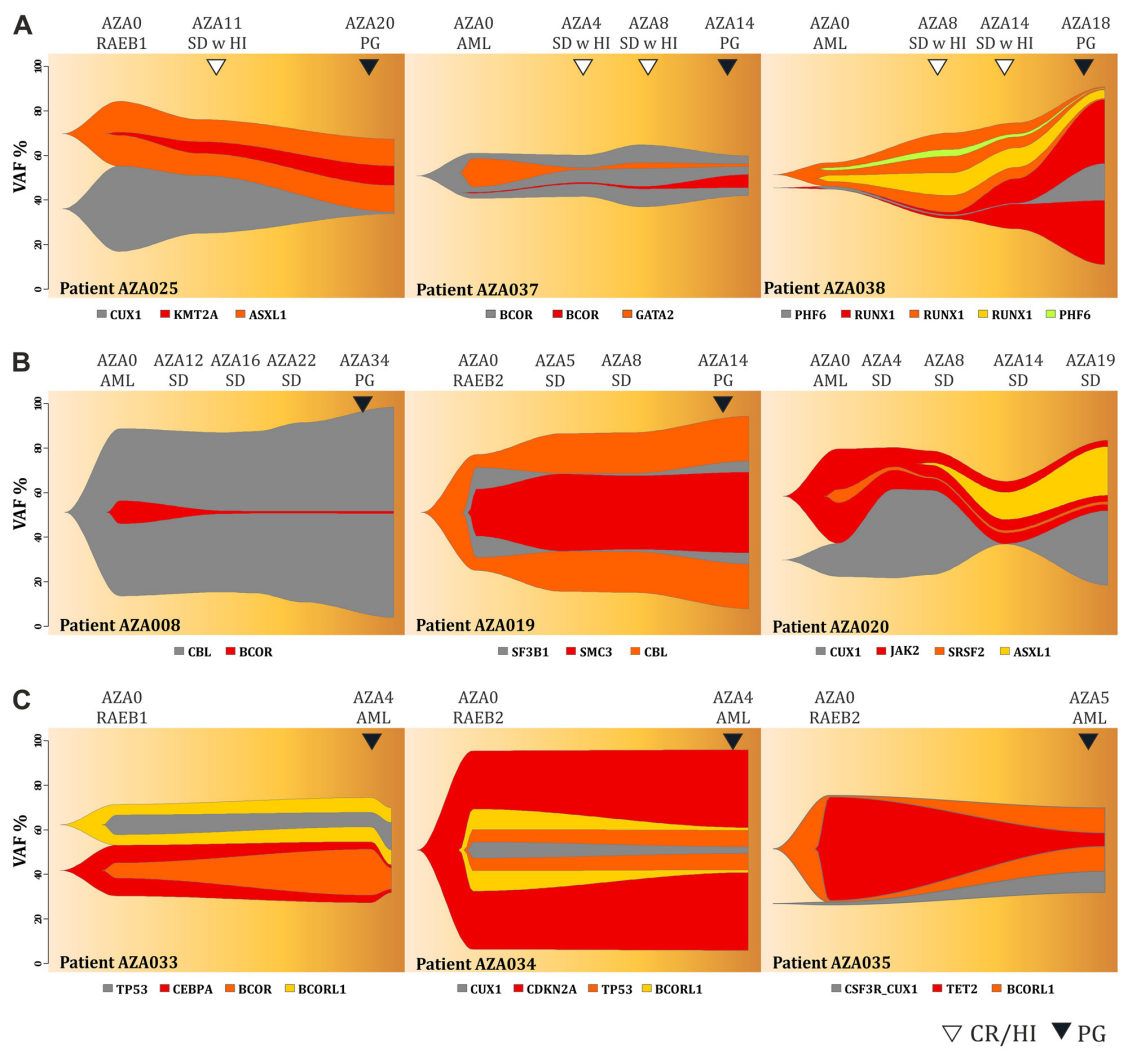
Fish plots of somatic variants detected in MDS patients with **(A)** stable disease achieving hematologic response, **(B)** stable disease without achieving hematologic response, **(C)** early progression on AZA therapy. Arrows indicate hematological improvement (HI, empty) and progression (PG, dark). Y-axis represents VAF (%) and X-axis is the time in weeks.

Next we focused on the profiles of MDS patients that partially responded to AZA by stable disease (SD) either with (Figure 4A) or without (Figure 4B) achieving hematology improvement (HI). The patients responding by ‘SD with HI’ displayed a decrease in VAF for some (but not all) variants (AZA025) at the time of HI while later during progression (PG) we again observed the variants reappearance or acquisition of new variants (AZA037, AZA038). Two patients with SD with HI had relatively stable mutation profile (AZA001, AZA031, see Supplementary Figure 1) with continuous increase of the mutation burden upon AML progression (AZA001, SF1). The mutation dynamics of SD patients with HI partly resembled the dynamics observed in the MDS patients responding by CR. In contrast, the ‘SD’ patients not achieving any HI (Figure 3B) had very stable and less dynamic mutation profiles throughout the course of AZA therapy. Similarly, when we studied mutation architecture of patients (N=4) that progressed early on AZA therapy, we noticed that the mutation changes were again very subtle and basically each mutation that a patient had at diagnosis was detected at the time of progression with almost identical VAF. These data support the notion that the therapy had negligible effect on the clonal pattern of the fast-progressing refractory disease in SD and PG patients (Figure 4C). Only limited clonal evolution was recorded in PG patients as exemplified by the patient AZA035 (Figure 4C). Complete set of Fishplots is shown in the Supplementary Figures 1 & 2. VAF for each variant and each patient is listed in the Supplementary Table 3. To conclude this part, we noted that patients reaching CR or HI (within SD) displayed substantial dynamics of mutation patterns indicating the AZA-imposed clonal selection process. In contrast, the changes of the mutation spectrum in patients with SD or PG were minimal (or for some variants the VAF steadily increased) thus implicating possible role of the detected variants mediating the AZA resistance.

### Statistical evaluation of the mutation dynamics

One of the main aims is to inspect the impact of mutated genes and their time-varying mutation dynamics during the AZA treatment on the overall survival and, consequently, their effect on the response duration on AZA. Since the VAF is measured at relatively distinct time points, a longitudinal data analysis cannot be directly applied. In order to discriminate the prognostic value of globally increasing mutations versus globally decreasing ones, signs of the regression slopes coming from a regression model on VAFs are taken into account. Moreover, to model the local time-dependent nature of mutation dynamics, time-varying characteristics such as the changing VAFs in time are considered. We utilize a joint model for the overall survival on the AZA and the response duration of the AZA. There are two types of covariates included in the joint model: constant over time (e.g., sex or globally decreasing/increasing status of mutation represented by a 0-1 indicator) and time varying (e.g., dichotomized VAF (5% level as a threshold) for a particular time point). The Cox proportional hazards (PH) model together with the Poisson count model provides a plausible framework, where the estimated parameters with the corresponding confidence intervals and p-values are shown in the Table 1.

**Table 1:** Fitted joint model for overall survival time and response duration on AZA

	Coefficient	SE	95% CI	Hazard ratio^*^ / Incidence rate^**^	95% CI	P-value
Cox PH model for OS time^*^				Hazard ratio	Score (logrank overall) test 0.0095	
Male vs Female	-0.906	0.453	-1.795, -0.018	0.404	0.166, 0.982	0.046
Age	-0.006	0.030	-0.065, 0.052	0.994	0.937, 1.054	0.833
CDKN2A mutated>5%	1.698	0.749	0.229, 3.166	5.461	1.258, 23.704	0.023
KDM6A mutated>5%	-1.598	0.632	-2.836, -0.360	0.202	0.059, 0.698	0.011
TP53 increasing	-1.489	0.673	-2.808, -0.169	0.226	0.060, 0.845	0.027
5q-	-1.496	0.495	-2.466, -0.525	0.224	0.085, 0.592	0.003

Poisson count model for Response duration^**^				Incidence rate	Likelihood ratio (overall) test <.0001	
Sex (Female as intercept)	0.324	0.887	-1.413, 2.062	1.383	0.243, 7.869	0.715
Male vs Female	1.141	0.179	0.791, 1.491	3.130	2.205, 4.442	<.0001
Age	0.006	0.012	-0.018, 0.029	1.006	0.982, 1.030	0.637
ASXL1 mutated>5%	1.137	0.249	0.650, 1.624	3.118	1.915, 5.075	<.0001
ASXL1 increasing	-1.665	0.452	-2.550, -0.779	0.189	0.078, 0.459	0.0002
BCOR mutated>5%	-1.079	0.337	-1.739, -0.420	0.340	0.176, 0.657	0.001
BCOR increasing	1.699	0.384	0.947, 2.451	5.467	2.577, 11.597	<.0001
BCORL1 increasing	-0.576	0.224	-1.015, -0.137	0.562	0.362, 0.872	0.010
CDKN2A mutated>5%	-2.750	0.481	-3.693, -1.808	0.064	0.025, 0.164	<.0001
CUX1 mutated>5%	1.981	0.361	1.274, 2.687	7.248	3.576, 14.691	<.0001
CUX1 increasing	-3.105	0.435	-3.957, -2.254	0.045	0.019, 0.105	<.0001
EZH2 mutated>5%	-3.304	0.529	-4.340, -2.267	0.037	0.013, 0.104	<.0001
KDM6A increasing	1.209	0.398	0.429, 1.989	3.350	1.536, 7.308	0.002
RUNX1 mutated>5%	0.863	0.192	0.486, 1.240	2.370	1.625, 3.454	<.0001
TP53 mutated>5%	1.733	0.537	0.680, 2.786	5.658	1.974, 16.211	0.001
TP53 increasing	-1.022	0.501	-2.004, -0.040	0.360	0.135, 0.960	0.041
5q-	1.254	0.185	0.892, 1.616	3.503	2.440, 5.031	<.0001

(A) Cox PH model for OS time; Score (logrank overall) test.0012.

(B) Poisson count model for Response duration; Likelihood ratio (overall) test <.0001.

SE indicates standard error; CI confidence interval; HR hazard ratio; IR incidence rate.

^*^ A positive (negative) coefficient estimate in the Cox PH model corresponds to a higher (lower) risk of death and thus on average a shorter (longer) OS time.

^**^ A positive (negative) coefficient estimate in the Poisson count model corresponds to higher (smaller) number of months of response duration.

**Figure 5:**
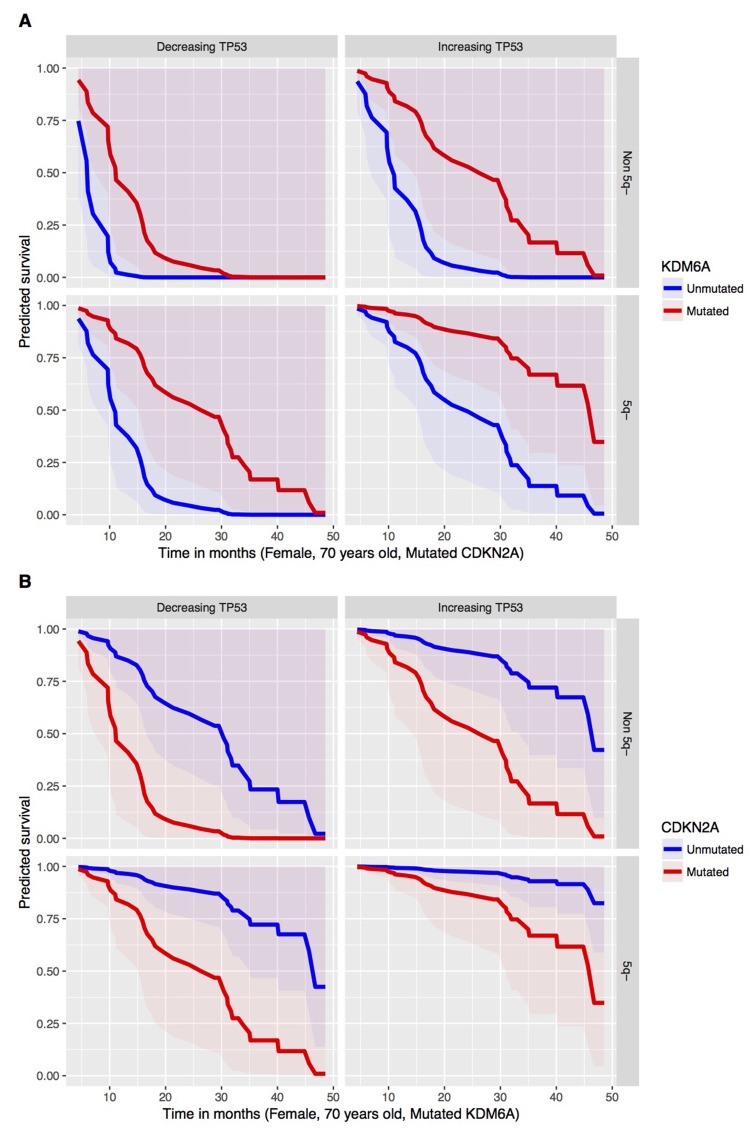
Predicted OS (y axis) in Time (x axis, Mo) in respect to the mutated (red) versus unmutated (blue) status of **(A)** *KDM6A*, **(B)** *CDKN2A* from the Cox PH model under conditions specified at the bottom of each survival plot for group with decreasing (left) or increasing (right) *TP53* mutation VAF dynamics.

From the joint fitted model, negative coefficient for the dynamics of *TP53* (p-value 0.027) can be noticed in case of the Cox PH part of the model. This means that a patient with a globally increasing VAF for *TP53* has lower risk of death. This highlights the possible effects of AZA therapy to at least partially control expansion of *TP53*-mutated clones thus supporting the notion that patients bearing certain *TP53* mutations can be treated with AZA to gain benefit in overall survival. Thus, one may judge that the increasing VAF corresponds to a tumor-static effect of the AZA and, therefore, yields longer surviving. Besides that, there is a significant effect on the overall survival caused by the following mutated genes: *CDKN2A* (p-value 0.023) and *KDM6A* (p-value 0.011). On one hand, presence of mutated *CDKN2A* tends to shorten the overall survival. On the other hand, *KDM6A* mutations yield lower risk of dying and, hence, longer overall survival. Presence of 5q- syndrome prior the development of high risk MDS (seen in about half of the patients in our cohort) provides significantly longer overall survival (p-value 0.003). Therefore, preceding 5q- syndrome can be viewed as good prognostic factor for patients entering AZA therapy. Furthermore, male patients from our cohort have in general longer overall survival (p-value 0.046). The predicted survival curves for the significant mutated genes in combination with the *TP53* dynamics (globally increasing vs decreasing mutation) and the 5q- syndrome are visualized in the Figure 5. It shows that preceding 5q- minus syndrome as a low risk MDS subtype provides favorable outcome for other mutations. In addition, *TP53* mutations that either increase VAF or decrease VAF on AZA have substantially different outcome supporting existence of additional mechanisms involved in the control of *TP53* mutant clones by AZA (see discussion). Finally, the Figure 5 provides more complex view when three variables (*TP53* mutation dynamics, preceding 5q- deletion, and *CDKN2A* mutation) are taken in account. Let us remark that the fitted joint model provides a multivariate approach, which can capture an interactive behavior of genes within a complex system. Therefore, it should not be surprising to obtain negative estimated parameters (coefficients) for some mutated genes or their dynamics.

**Figure 6:**
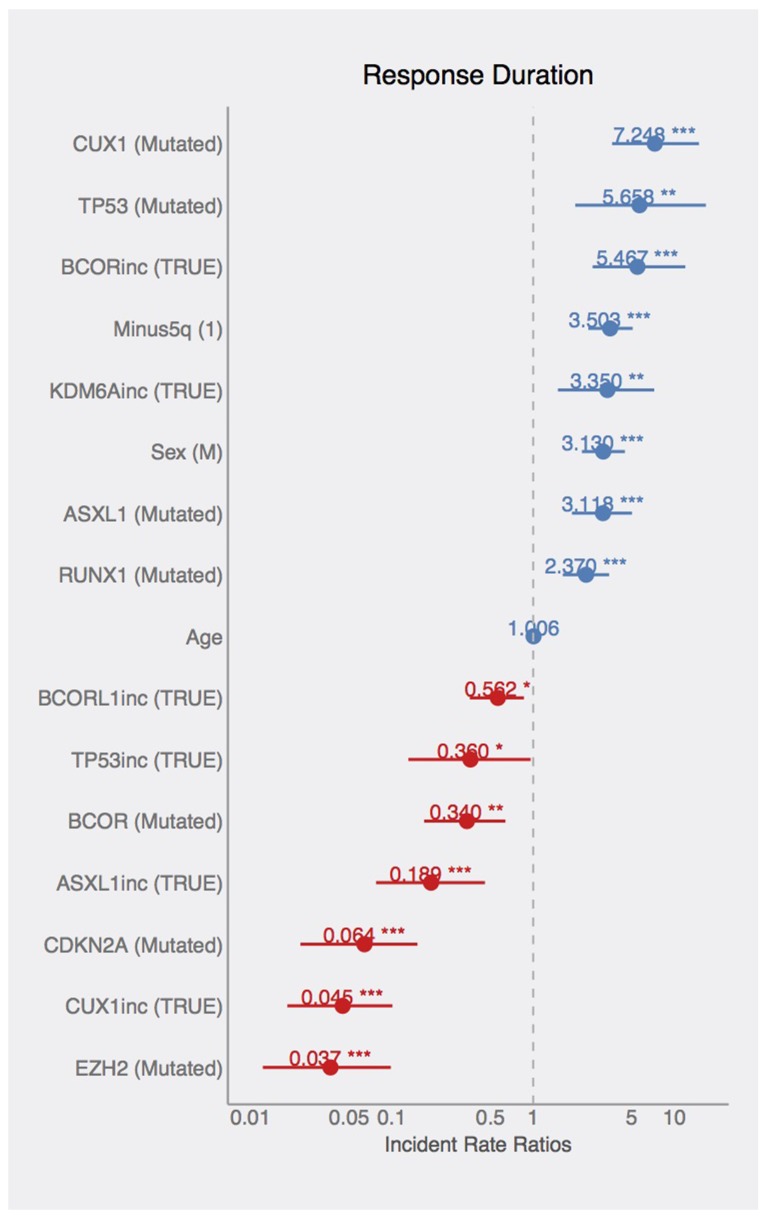
Effects of mutations on AZA-response duration Coefficients in the Poisson count model documenting the estimated incident rates. Effects of listed variables on response duration (blue=increased, red=decreased RD).

The second part of the joint model represents the response duration on AZA. There is a significant effect of sex on the response duration, where male patients tend to have longer response duration. The significant effects of mutated or dynamically changing (increasing or decreasing pattern of) allelic burden of *ASXL1, BCOR, BCORL1, CDKN2A, CUX1, EZH2, KDM6A, RUNX1*, and *TP53* genes were also relieved with respect to the response duration. Mutated *ASXL1, CUX1, RUNX1*, and *TP53* (VAF above 5%) are found in patients with longer response duration (again indicating that these variants-bearing clones can be controlled by AZA or, alternatively, these mutations occur in longer-responding patients efficiently controlled by AZA), whereas *BCOR, CDKN2A*, and *EZH2* mutations significantly shorten the response duration and thus the effect of AZA to control the mutation-bearing clones appears to be lower. Such control however is not guaranteeing the response as the globally increasing VAF for *ASXL1, BCORL1, CUX, TP53* and globally decreasing VAFs for *BCOR, KDM6A* mean shorter response duration. In addition, the patients with 5q- syndrome have significantly longer response duration supporting the above stated notion of preceding 5q- low risk MDS as positive predictive factor. The estimated incidence rates (statistically significant from constant 1) are displayed in the Figure 6.

## DISCUSSION

In this study we present the NGS approach using high accuracy detection of the pathogenic MDS variants via serial sampling of 38 high risk MDS patients treated with AZA (Figures 1,2). Interestingly, a marked decrease of VAF upon AZA was observed in patients achieving CR while minor changes in VAF precluded minimal response often seen in PG and SD patients (Figures 3,4). Although our study indicates that AZA influences overall variants’ dynamics (Figures 5,6 and Table 1) only some of the individual mutation dynamics during AZA therapy have significant impact on the clinical outcome. While several studies suggested that MDS patients in progression may evolve new mutations and lose some of the clonal architecture detected at preceding stages [10] we suggest that progression on AZA is rather associated with proliferation of original clones with variants detected shortly prior AZA therapy. This contention is supported by other study indicating that responders to hypomethylating agents bear certain gene mutations [7] further implicating that some variants may in turn encode clonal tolerance to AZA. Besides genetic factors one could not exclude also epigenetic factors to mediate AZA tolerance (of the original clone) responsible for the clinical relapse possibly also involving additional variables not herein studied such as the global effects of DNA methylation [11] or expression of specific non-coding RNAs [12] that can add to complexity of the AZA sensitivity versus AZA resistance.

The tool for detecting the dynamics of somatic mutations was the TruSight Myeloid Panel that contains 54 gene regions with previously documented mutation recurrence in 439 patients [13]. The TruSight Myeloid Panel is a collection of frequently mutated target genes related to myeloid hematology disorders including MDS and AML and was designed by a consortium of the recognized experts. Many of the observed mutations were indeed previously associated with the clinical outcome including *TP53*, *RUNX1*, *ASXL1*, *EZH2* [14] or *TET2* and *ASXL1* [7]. However, not only presence or absence of the mutation (above arbitrary cutoff 5% VAF) matters but also the dynamics of such variant may matter. The variant dynamics observed in our study indeed associated with the major clinical outcomes: response duration and overall survival. While there exist gene variants that are not fully extinguished by AZA there are variants that become depleted especially upon achievement of CR (Figure 3). Interestingly, the patients with *TP53* mutations consistently increasing their VAF on AZA therapy were however characterized by significantly prolonged survival while their response duration was relatively short. This implicates that the response to AZA was neutral (stable disease), which precludes some potential of AZA in controlling the growth of the *TP53*-bearing clones. Recently, *TP53* mutations were shown as the only molecular signature predictive of a CR in the therapy with another DNMTi, Decitabine [15]. It is therefore possible that demethylation therapy with AZA have also some inhibitory effects in the therapeutic control of the *TP53*-mutated clones. It is however possible that these *TP53*-mutated clones may bear less aggressive variants. One can envision that AZA at standard dose is unable to control *TP53*-mutated clones indefinitely and over time their allelic burden accumulates. Relatively slow rate of progression of the *TP53*-mutated patients during AZA therapy may be still relatively well tolerated by the patients thus guaranteeing a survival benefit. It is also an important point that the mutations operate with other mutations in concert and thus higher occurrence of preceding 5q- aberration may have added to selection (during clonal evolution) of distinct *TP53* mutations (observed in up to 31% of 5q- patients) [16] that are not as aggressive and still may provide certain cell-survival benefit for maturing blood elements to maintain residual hematopoiesis while preventing rapid progression to MDS/AML.

Additionally, the patients with IPSS-defined high risk MDS are however already at very high risk of dying so the mutation in *TP53* may not represent the most critical risk factor thus supporting the potential roles for other predictive factors of AZA response such as the *CDKN2A* and *KDM6A* variants seen in our study (Figure 6, Table 1). Interestingly, while variants of *EZH2* were previously shown to associate with adverse clinical outcome in MDS [17] and were in our study predictive of adverse response to AZA, the variants in *CDKN2A* were not yet previously considered (see Figure 6, Table 1). Nevertheless, there likely exist additional regions (not herein studied) whose mutations may affect progression to AML and whose analysis will require additional global-NGS approaches. Variants potentially involved in the AML progression are highlighted in the Supplementary Table 3.

To conclude, utilizing NGS at the restaging BM analysis represents systematic approach for analyzing molecular response to the AZA therapy, and moreover, it provides newly not yet considered predictive features. We think that the serial analyses of MDS patients by NGS or digital PCR (that is also capable to determine precisely the VAF) at restaging periods will further validate our herein presented data to further strengthen the impact of mutation dynamics during AZA therapy.

## MATERIALS AND METHODS

### Patient samples

MDS patients (Supplementary Table 1, Figure 1) with Int-2/high IPSS risk or MDS/AML with less than 30% myeloblasts in BM cytology were treated by AZA in 1-month cycles until PG with 75 mg/m^2^ Vidaza (Celgene) in 5+2+2 regimen. Response criteria for CR, HI, or SD were used exactly as described elsewhere [18]. Marrow and cytogenetic responses were monitored as well during each bone marrow (BM) restaging period every 4 months (see Supplementary Table 3). BM samples representing residua of those used for routine diagnostics were cryopreserved in liquid nitrogen with patients’ agreement to analyze the DNA content. Patient samples were collected in years 2010-2016 following the written informed consent based on the Helsinki declaration, and approved by the Ethics Committee of the General Hospital Prague. The cohort contained 38 patients that provided in total 97 BM samples; most of them were magnetically separated into T-cell CD3^+^ and the myeloid CD3-negative fractions (MACS, Miltenyi, Germany). AZA therapy was applied for median 14 cycles (range 4-34) to patients (median 70 year-old) not indicated for allogenic BM transplantation. Treatment responses were as follows: CR (11 patients, 29%), SD with HI (11, 29%), SD (12, 32%), and finally no response with PG on AZA (4, 10%). The patient cohort was biased in patient selection as it contains more AZA responders and less early-progressing patients. OS on AZA was as follows: CR >12 Mo (29.6 Mo), CR<12 Mo (17.6 Mo), SD w HI (31 Mo), SD (32.4 Mo) and PG (6.1 Mo). 25 (66%) patients progressed to AML. Median OS range was 4-48 months; 5 patients are currently alive with the follow-up exceeding four years.

### Sequencing

DNA was isolated using DNeasy Blood and Tissue Kit (Qiagen, Hilden, Germany) and further processed using TruSight Myeloid Panel Kit (Illumina, San Diego, USA) according to the manufacturer’s recommendations. Illumina Hi-Seq and Illumina Mi-Seq instruments were used to obtain sequence data. The panel focuses on ∼141 kb of genomic content, consisting of 568 amplicons of ∼250 bp in length designed against the human NCBI37/hg19 reference genome. The oligo-pool targets 15 full genes (exons only) plus exonic hotspots of an additional 39 genes, providing nearly 100% coverage of all targeted regions. The uniform coverage of the target regions enables > 500× coverage for > 95% of amplicons at > 5,000 × mean coverage. Reproducibility of the sequencing was very high and majority of samples had very similar VAF and contained same gene variants. Although the sequencing runs were quite homogeneous there existed a rare example with lower overlap (∼60%) of the detected variants. Average amplicon coverage was > 1000. For each patient we have collected multiple bone marrow samples during the course of the disease, see Figure 1. CD3^-^ fraction was sequenced for each sample. For each patient we have sequenced at least one CD3^+^ fraction. We have sequenced at least one sample per patient twice including two separate sequencing library preparations. Moreover, we use age-matched controls and cord blood samples as internal controls. Somatic variants were primarily detected using CD3^-^ fraction, while duplicate samples and CD3^+^ fraction data were used for control and filtering. We use the term sequencing unit to refer to a particular sequenced fraction or one sequencing library from a repeatedly-sequenced sample. By the VAF we mean the proportion of sequencing reads that harbor a variant to the total number of reads in a given region for given sequencing unit. Note that this number is related to the proportion of cells that contain the variant to the total number of cells in the whole cell population of particular sequencing unit.

### Processing of sequencing data

FASTQ files produced by the sequencer were processed by custom pipeline. Initial quality control was performed by means of FastQC (http://www.bioinformatics.babraham.ac.uk/projects/fastqc). After sequencing adaptor trimming and low quality regions removal by cutadapt, reads were aligned to the human genome HG19 using bwa mem (bwa version: 0.7.15-r1140). Subsequently, bam files were processed by GATK IndelRealigner and primer sequences were removed from bam files.

### Variant detection

We used two variant calling tools (*FreeBayes* and *samtools mpileup*) [19] to detect SNVs and InDels. We then annotated detected variants using dbSNP and COSMIC databases. Criteria for selecting reported variants were as follows. We use data from the union of variant detections performed using *FreeBayes* and *samtools mpileup* with default set of parameters to these programs. For each sequencing unit from MDS patient, we computed VAF and reported only the variants with VAF value that was greater by 20 percentage points than the highest VAF for the variant in an internal control. Mutation was considered somatic and reported if not detected in the CD3^+^ fraction and if successfully detected in a duplicate library sample. Putative pathogenic variants that markedly affected protein structure also excluding polymorphisms were further filtered by experienced biologist. The arbitrary VAF was set at 5% as inspired by other studies [9, 10].

### Data visualization

We made clusters of variants that had a similar evolution during the course of the disease. Majority of clusters in our dataset was formed by a single variant. By visual inspection of the data we made predictions regarding clonal architecture. Based on this data, we visualized clonal architecture using fishplot package (https://bmcgenomics.biomedcentral.com/articles/10.1186/s12864-016-3195-z).

### Statistical analysis

Multivariate joint model consisting of the Cox PH and the Poisson count models including the variant dynamics score utilized the logrank score and the likelihood ratio tests. Statistics is in detail provided also within the last Results’ section.

## SUPPLEMENTARY MATERIALS FIGURE AND TABLES







## Abbreviations

MDS: myelodysplastic syndrome
AML: acute myeloid leukemia
AZA: azacitidine
DNMTi: DNA methyltransferase inhibitor
ST: stable
DEC: decreasing
INC: increasing
U: U-shape
RD: response duration
OS: overall survival
VAF: variant allele frequency
SD: stable disease
HI: hematology improvement
PG: progressed disease
CR: complete remission
NGS: next generation sequencing

## References

[1] Genovese G, Kahler AK, Handsaker RE, Lindberg J, Rose SA, Bakhoum SF, Chambert K, Mick E, Neale BM, Fromer M, Purcell SM, Svantesson O, Landen M (2014). Clonal hematopoiesis and blood-cancer risk inferred from blood DNA sequence. N Engl J Med.

[2] Will B, Zhou L, Vogler TO, Ben-Neriah S, Schinke C, Tamari R, Yu Y, Bhagat TD, Bhattacharyya S, Barreyro L, Heuck C, Mo Y, Parekh S (2012). Stem and progenitor cells in myelodysplastic syndromes show aberrant stage-specific expansion and harbor genetic and epigenetic alterations. Blood.

[3] Fenaux P, Mufti GJ, Hellstrom-Lindberg E, Santini V, Gattermann N, Germing U, Sanz G, List AF, Gore S, Seymour JF, Dombret H, Backstrom J, Zimmerman L (2010). Azacitidine prolongs overall survival compared with conventional care regimens in elderly patients with low bone marrow blast count acute myeloid leukemia. J Clin Oncol.

[4] Kantarjian H, Oki Y, Garcia-Manero G, Huang X, O'Brien S, Cortes J, Faderl S, Bueso-Ramos C, Ravandi F, Estrov Z, Ferrajoli A, Wierda W, Shan J (2007). Results of a randomized study of 3 schedules of low-dose decitabine in higher-risk myelodysplastic syndrome and chronic myelomonocytic leukemia. Blood.

[5] Fenaux P, Gattermann N, Seymour JF, Hellstrom-Lindberg E, Mufti GJ, Duehrsen U, Gore SD, Ramos F, Beyne-Rauzy O, List A, McKenzie D, Backstrom J, Beach CL (2010). Prolonged survival with improved tolerability in higher-risk myelodysplastic syndromes: azacitidine compared with low dose ara-C. Br J Haematol.

[6] Craddock C, Quek L, Goardon N, Freeman S, Siddique S, Raghavan M, Aztberger A, Schuh A, Grimwade D, Ivey A, Virgo P, Hills R, McSkeane T (2013). Azacitidine fails to eradicate leukemic stem/progenitor cell populations in patients with acute myeloid leukemia and myelodysplasia. Leukemia.

[7] Bejar R, Lord A, Stevenson K, Bar-Natan M, Perez-Ladaga A, Zaneveld J, Wang H, Caughey B, Stojanov P, Getz G, Garcia-Manero G, Kantarjian H, Chen R (2014). TET2 mutations predict response to hypomethylating agents in myelodysplastic syndrome patients. Blood.

[8] Jung SH, Kim YJ, Yim SH, Kim HJ, Kwon YR, Hur EH, Goo BK, Choi YS, Lee SH, Chung YJ, Lee JH (2016). Somatic mutations predict outcomes of hypomethylating therapy in patients with myelodysplastic syndrome. Oncotarget.

[9] Tobiasson M, McLornan DP, Karimi M, Dimitriou M, Jansson M, Ben Azenkoud A, Jadersten M, Lindberg G, Abdulkadir H, Kulasekararaj A, Ungerstedt J, Lennartsson A, Ekwall K (2016). Mutations in histone modulators are associated with prolonged survival during azacitidine therapy. Oncotarget.

[10] Pellagatti A, Roy S, Di Genua C, Burns A, McGraw K, Valletta S, Larrayoz MJ, Fernandez-Mercado M, Mason J, Killick S, Mecucci C, Calasanz MJ, List A (2016). Targeted resequencing analysis of 31 genes commonly mutated in myeloid disorders in serial samples from myelodysplastic syndrome patients showing disease progression. Leukemia.

[11] Qin T, Castoro R, El Ahdab S, Jelinek J, Wang X, Si J, Shu J, He R, Zhang N, Chung W, Kantarjian HM, Issa JP (2011). Mechanisms of resistance to decitabine in the myelodysplastic syndrome. PLoS One.

[12] Butrym A, Rybka J, Baczynska D, Poreba R, Kuliczkowski K, Mazur G (2016). Clinical response to azacitidine therapy depends on microRNA-29c (miR-29c) expression in older acute myeloid leukemia (AML) patients. Oncotarget.

[13] Bejar R, Stevenson K, Abdel-Wahab O, Galili N, Nilsson B, Garcia-Manero G, Kantarjian H, Raza A, Levine RL, Neuberg D, Ebert BL (2011). Clinical effect of point mutations in myelodysplastic syndromes. N Engl J Med.

[14] Bejar R, Stevenson KE, Caughey B, Lindsley RC, Mar BG, Stojanov P, Getz G, Steensma DP, Ritz J, Soiffer R, Antin JH, Alyea E, Armand P (2014). Somatic mutations predict poor outcome in patients with myelodysplastic syndrome after hematopoietic stem-cell transplantation. J Clin Oncol.

[15] Chang CK, Zhao YS, Xu F, Guo J, Zhang Z, He Q, Wu D, Wu LY, Su JY, Song LX, Xiao C, Li X (2017). TP53 mutations predict decitabine-induced complete responses in patients with myelodysplastic syndromes. Br J Haematol.

[16] Stengel A, Kern W, Haferlach T, Meggendorfer M, Haferlach C (2016). The 5q deletion size in myeloid malignancies is correlated to additional chromosomal aberrations and to TP53 mutations. Genes Chromosomes Cancer.

[17] Nikoloski G, Langemeijer SM, Kuiper RP, Knops R, Massop M, Tonnissen ER, van der Heijden A, Scheele TN, Vandenberghe P, de Witte T, van der Reijden BA, Jansen JH (2010). Somatic mutations of the histone methyltransferase gene EZH2 in myelodysplastic syndromes. Nat Genet.

[18] Cheson BD, Greenberg PL, Bennett JM, Lowenberg B, Wijermans PW, Nimer SD, Pinto A, Beran M, de Witte TM, Stone RM, Mittelman M, Sanz GF, Gore SD (2006). Clinical application and proposal for modification of the international working group (IWG) response criteria in myelodysplasia. Blood.

[19] Li H, Handsaker B, Wysoker A, Fennell T, Ruan J, Homer N, Marth G, Abecasis G, Durbin R (2009). 1000 Genome Project Data Processing Subgroup. The sequence alignment/map format and samtools. Bioinformatics.

