# Supplementary material for: Somatic mutation dynamics in MDS patients treated with azacitidine indicate clonal selection in patients-responders
Source: Oncotarget. 2017 Dec 6;8(67):111966–78. doi: 10.18632/oncotarget.22957 (PMC5762372; doi:10.18632/oncotarget.22957)
Supplement: Supplementary file 1 [file oncotarget-08-111966-s001.pdf]

Somatic mutation dynamics in MDS patients treated with azacitidine indicate clonal selection in patients-responders

SUPPLEMENTARY MATERIALS

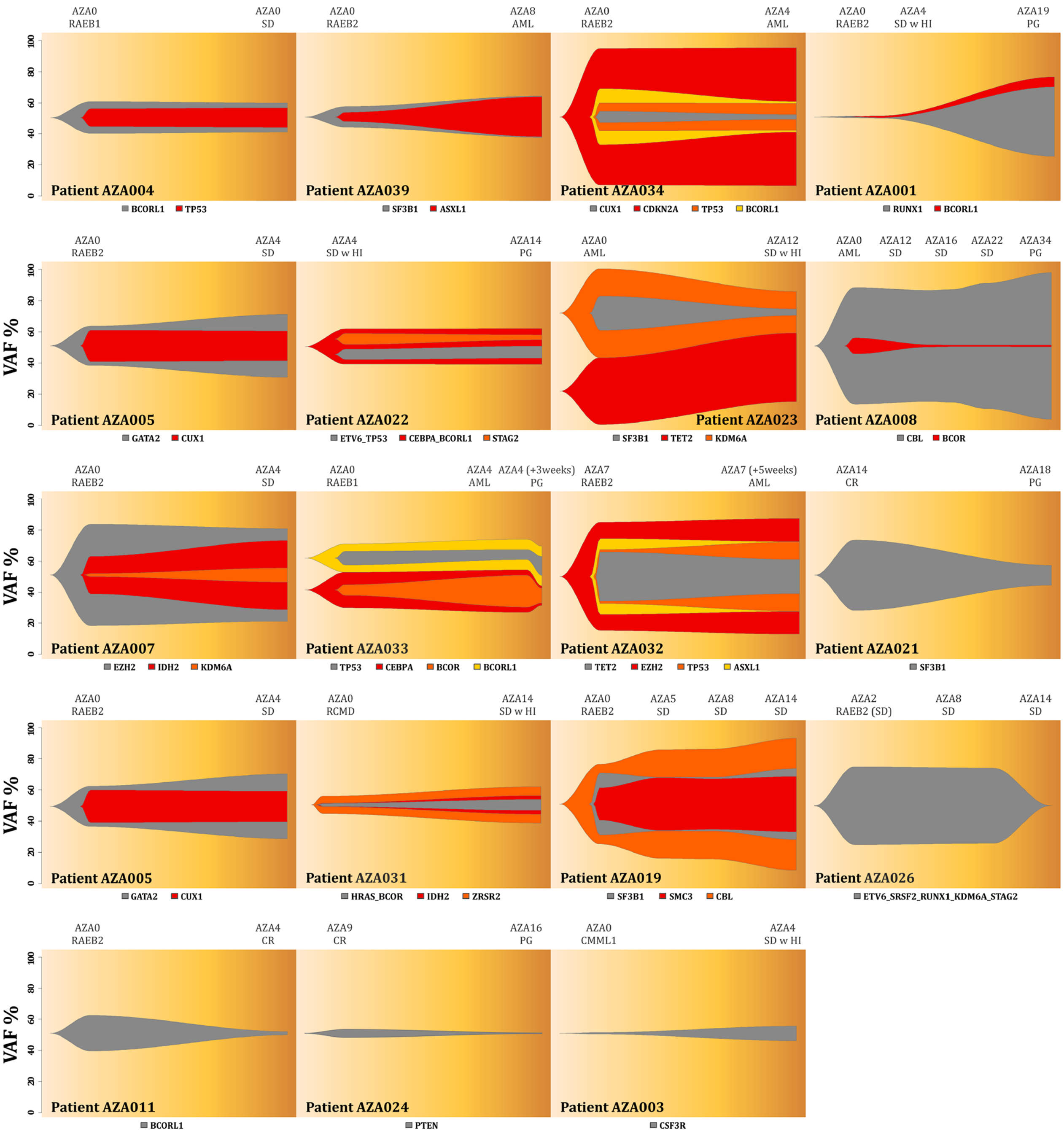

Supplementary Figure 1: Fish plots of somatic variants with stable profiles detected in MDS patients.

Supplementary Table 1: Clinical data of the MDS cohort indicating (from top): demographics, MDS classification, IPSS-R, cytogenetics, selected BM and PB findings, and finally the responses

**Demographics**		
Age (median, range)	70	57-82
Sex (number, %)		
Female	20	53%
Male	18	47%
**MDS classification (number, %)**		
RA	0	0%
RARS	0	0%
RCMD	1	3%
RAEB1	13	34%
RAEB2	17	45%
MDS/AML	5	13%
CMML	2	5%
**IPSS-R risk group (number, %)**		
Very low	0	0%
Low	1	3%
Intermediate	14	37%
High	18	47%
Very high	5	13%
**Cytogenetics risk group (number, %)**		
Very good	0	0%
Good	26	68%
Intermediate	5	13%
Poor	4	11%
Very poor	3	8%
**Karyotype (number, %)**		
Normal	16	42%
Abnormal	22	58%
**Clinical characteristics (median, range)**		
BM-Blasts [%]	10	2-26
PB-Hemoglobin [g/L]	91	62-128
PB-ANC [x10^9/L]	1.24	0.01-11.6
PB-Platelets [x10^9/L]	80	2-576
**Outcome (number, %)**		
CR	11	29%
SD with HI	11	29%
SD without HI	12	10,5 %
primary PG	4	31,5 %
AML transformation (total)	24	63%

Supplementary Table 2: Complete list of detected variants. Patients' codes are in the 1st column. Responses to AZA are listed in the 2nd column: PG progression, SD stable disease, SD w HI, CR: short (<12 Mo) and long (>12Mo). Mutation dynamics is listed in the 3rd column (VAF INC= increasing more than 2-fold, DEC=decreasing more than 2-fold, complex dynamics: INC-DEC & DEC-INC, ST= stable). Gene symbols, gene localization, amino acid change (AA), and COSMIC assembly (or NA= not assembled) as indicated.

See Supplementary File 1

Supplementary Table 3: List of all variants and clinical and laboratory data. Codes of AZA-treated patients, their OS (Mo), IPSS-R, sample codes, % BM myeloblasts (MB), cycles of AZA, cytogenetic data, occurrence of AML progression together with gene mutations and their VAF accompanied by reads numbers are shown.

See Supplementary File 2

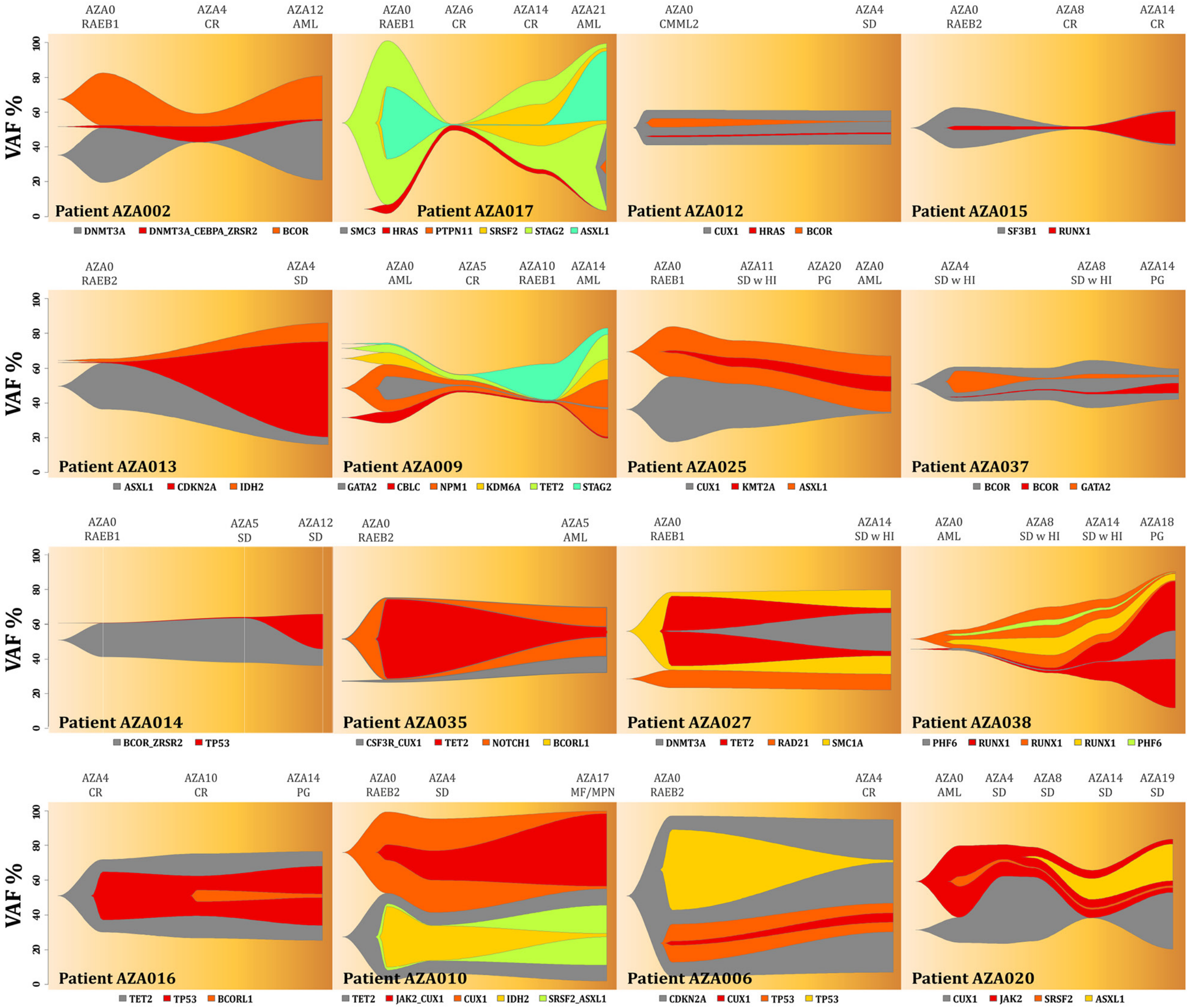

Supplementary Figure 2: Fish plots of somatic variants with highly unstable and variable profiles detected in MDS patients.
