# Supplementary material for: Somatic mutation dynamics in MDS patients treated with azacitidine indicate clonal selection in patients-responders
Source: Oncotarget. 2017 Dec 6;8(67):111966–78. doi: 10.18632/oncotarget.22957 (PMC5762372; doi:10.18632/oncotarget.22957)
Supplement: Supplementary file 2 [file oncotarget-08-111966-s002.docx]

**Supplementary Table 2: Complete list of detected variants**. Patients’ codes are in the 1^st^ column. Responses to AZA are listed in the 2^nd^ column: PG progression, SD stable disease, SD w HI, CR: short (<12 Mo) and long (>12Mo). Mutation dynamics is listed in the 3^rd^ column (VAF INC= increasing more than 2-fold, DEC=decreasing more than 2-fold, complex dynamics: INC-DEC & DEC-INC, ST= stable). Gene symbols, gene localization, amino acid change (AA), and COSMIC assembly (or NA= not assembled) as indicated.

| **PATIENT CODE** | **CLINICAL OUTCOME** | **Dynamics** | **GENE** | **Variant** | **AA** | **COSMIC** |
| --- | --- | --- | --- | --- | --- | --- |
| AZA001 | SD w HI | INC | BCORL1 | chrX:129148264_T/A | p.Phe506Ile | NA |
| AZA001 | SD w HI | INC | RUNX1 | chr21: 36252865 | p.Arg166Gln | COSM36055 |
| AZA002 | CR long | DEC-INC | BCOR | chrX: 39933593 | p.Ser336fs | COSM4385749 |
| AZA002 | CR long | DEC-INC | RUNX1 | chrX:15808619_AT/AG | p.Met1? | NA |
| AZA002 | CR long | INC-DEC | CEBPA | chr19:33792335_TCC/TC | p.Glu364fs | NA |
| AZA002 | CR long | INC-DEC | DNMT3A | chr2:25464475_T/C | p.Lys680Glu | NA |
| AZA002 | CR long | INC-DEC | ZRSR2 | chr21:36206748_TGG/TGGG | p.His255fs | NA |
| AZA003 | SD w HI | INC | CSFR3 | chr1:36932464_T/C | p.Met696Val | NA |
| AZA004 | SD w HI | ST | BCORL1 | chrX: 129147373 | p.Gly209Ser | COSM5019165 |
| AZA004 | SD w HI | ST | TP53 | chr17: 7578268 | p.Leu194Arg | COSM117650 |
| AZA005 | SD | ST | CUX1 | chr3:128200124_TG/CA | p.Gln394Trp | NA |
| AZA005 | SD | ST | GATA2 | chr7:101870796_GA/GC | p.Asp1105Ala | NA |
| AZA006 | CR short | DEC | TP53 | chr17: 7578190 | p.Tyr220Cys | COSM99719 |
| AZA006 | CR short | ST | CDKN2A | chr9: 21971137 | p.Asp74Ala | COSM4163710 |
| AZA006 | CR short | ST | CUX1 | chr7:101844790_TCACCATCCTCA/TCCCCATCCTCC | p.ThrIleLeuThr750ProIleLeuPro | NA |
| AZA006 | CR short | ST | TP53 | chr17:7572977_AGGTAGAC/GGGGAGAC | p.ThrSer377ProPro | NA |
| AZA007 | SD | INC | KDM6A | chrX:44945122_A/G | p.Asn1201Ser | NA |
| AZA007 | SD | ST | EZH2 | chr7: 148512096 | p.Cys528Arg | COSM4384278 |
| AZA007 | SD | ST | IDH2 | chr15: 90631934 | p.Arg140Gln | COSM41590 |
| AZA008 | SD | DEC | BCOR | chrX:39933593_A/G | p.Ser336Pro | NA |
| AZA008 | SD | ST | CBL | chr11: 119148930 | p.Cys384Arg | COSM34057 |
| AZA009 | CR short | DEC | CBLC | chr19:45297500_CT/CG | p.Leu442Arg | NA |
| AZA009 | CR short | DEC | GATA2 | chr3:128200106 | p.Lys399Glu | NA |
| AZA009 | CR short | DEC-INC | KDM6A | chrX:44922984_CAC/CAAC | p.Thr668fs | NA |
| AZA009 | CR short | DEC-INC | NPM1 | chr5:170837552_TGGAGGA/TGCTTTTCCCA | p.Trp290fs | NA |
| AZA009 | CR short | DEC-INC | TET2 | chr4:106180886_TCC/TCCC | p.Arg1307fs | NA |
| AZA009 | CR short | INC | STAG2 | chrX:123195616_TTTAG/TG | splicing variant | NA |
| AZA009 | CR short | ST | BCORL1 | chrX: 129147373 | p.Gly209Ser | COSM5019165 |
| AZA010 | SD | DEC | IDH2 | chr15: 90631934 | p.Arg140Gln | COSM41590 |
| AZA010 | SD | DEC-INC | SRSF2 | chr17:74732959_GGGC/TGGG | p.ArgPro94ProHis | NA |
| AZA010 | SD | INC | CUX1 | chr7:101740676_CTCCAG/CTCCATCCAG | p.Gln113fs | NA |
| AZA010 | SD | INC | JAK2 | chr9: 5073770 | p.Val617Phe | COSM12600 |
| AZA010 | SD | ST | ASXL1 | chr20:31022441 | p.Gly646fs | COSM4170082 |
| AZA010 | SD | ST | ATRX | chrX:76856021 | p.Asn1860Ser | COSM5001991 |
| AZA010 | SD | ST | CUX1 | chr7: 101758502 | p.Arg219Gln | COSM4409340 |
| AZA010 | SD | ST | TET2 | chr4: 106155248 | p.Asp51fs | NA |
| AZA011 | CR short | DEC | BCORL1 | chrX: 129147373 | p.Gly209Ser | COSM5019165 |
| AZA012 | SD | DEC | BCOR | chrX:39930384_T/C | p.Glu1027Gly | NA |
| AZA012 | SD | INC | HRAS | chr11:533772_TGG/TG | p.Gln95fs | NA |
| AZA012 | SD | ST | CUX1 | chr7:101870796_GA/GC | p.Asp1105Ala | NA |
| AZA013 | SD | DEC | ASXL1 | chr20: 31022441 | p.Gly646fs | COSM4170082 |
| AZA013 | SD | INC | CDKN2A | chr9: 21970916 | p.Ala148Thr | COSM3774362 |
| AZA013 | SD | INC | IDH2 | chr15: 90631835 | p.His173Pro | COSM3999681 |
| AZA014 | SD | DEC | ZRSR2 | chrX:15808655_C/A | p.Pro13Thr | NA |
| AZA014 | SD | INC | TP53 | chr17: 7572991 | p.Lys373Arg | COSM3727817 |
| AZA014 | SD | ST | CDKN2A | chr9: 21971137 | COSM4163710 | p.Asp74Ala |
| AZA015 | CR long | DEC-INC | SF3B1 | chr2: 198267360 | p.Lys666Thr | COSM131556 |
| AZA015 | CR long | INC | RUNX1 | chr21:36206722_G/A | p.Gln264* | NA |
| AZA016 | CR short | INC | BCORL1 | chrX:129148327_C/A | p.Pro527Thr | NA |
| AZA016 | CR short | ST | TET2 | chr4: 106196829 | p.Leu1721Trp | COSM5020013 |
| AZA016 | CR short | ST | TP53 | chr17: 7578190 | p.Tyr220Cys | COSM99719 |
| AZA017 | CR long | DEC | HRAS | chr11:533895_T/C | p.Asp54Gly | NA |
| AZA017 | CR long | DEC-INC | ASXL1 | chr20:31022877_GAATGTGAGTCTGGCACCACTTC/GAATGTGAGTCTGGCACCACTTAATGTGAGTCTGGCACCACTTC | p.Thr794_Ser795insTerCysGluSerGlyThrThr | NA |
| AZA017 | CR long | DEC-INC | SRSF2 | chr17: 74732935 | p.Pro95_Arg102del | COSM1318446 |
| AZA017 | CR long | DEC-INC | STAG2 | chrX:123159689_G/A | splicing | NA |
| AZA017 | CR long | INC | PTPN11 | chr12: 112888162 | p.Gly60Arg | COSM13010 |
| AZA017 | CR long | INC | SMC3 | chr10:112356192_G/C | p.Gly667Ala | NA |
| AZA018 | SD | NA | NA | NA | NA | NA |
| AZA019 | SD | ST | CBL | chr11: 119149246 | p.Phe418Leu | COSM34078 |
| AZA019 | SD | ST | SF3B1 | chr2: 198267361 | p.Lys666Gln | COSM132950 |
| AZA019 | SD | ST | SMC3 | chr10:112344020_A/G | p.Arg391Gly | NA |
| AZA020 | SD | DEC | SRSF2 | chr17:74733211_T/C | p.Glu11Gly | NA |
| AZA020 | SD | DEC-INC | JAK2 | chr9: 5073770 | p.Val617Phe | COSM12600 |
| AZA020 | SD | INC | ASXL1 | chr20: 31022286 | p.Tyr591fs | COSM4169775 |
| AZA020 | SD | INC | CUX1 | chr7:101844812_TCTGTCCA/TCTGTCCC | p.Thr759Pro | NA |
| AZA021 | CR long | DEC | SF3B1 | chr20: 198266834 | p.Lys700Glu | COSM84677 |
| AZA022 | SD v HI | DEC | STAG2 | chrX:123227945_T/C | p.Ile1219Thr | NA |
| AZA022 | SD w HI | ST | BCORL1 | chrX: 129147373 | p.Gly209Ser | COSM5019165 |
| AZA022 | SD w HI | ST | CEBPA | chr19:33792348_GCAGGCGGTCATTGTCAC/GC | p.Asp355fs | NA |
| AZA022 | SD w HI | ST | ETV6 | chr12:12022755_CAA/CGA | p.Lys288Glu | NA |
| AZA022 | SD w HI | ST | STAG2 | chrX:123197751_TAC/TAAC | p.Thr626fs | NA |
| AZA022 | SD w HI | ST | TP53 | chr17: 7578268 | p.Leu194Arg | COSM117650 |
| AZA023 | SD v HI | DEC | SF3B1 | CHR2: 198266834 | p.Lys700Glu | COSM84677 |
| AZA024 | CR short | DEC | PTEN | CHR10: 89692848 | p.Trp284* | COSM5157 |
| AZA025 | SD v HI | DEC | CUX1 | Chr7: 101844834_GCCCA/GCCCC | p.Thr764Pro | NA |
| AZA025 | SD w HI | INC | MLL(KDMT2A) | chr11:118307393_T/C | p.Ser56Pro | NA |
| AZA025 | SD w HI | ST | ASXL1 | chr20: 31022441 | p.Gly646fs | COSM4170082 |
| AZA026 | SD | DEC | ETV6 | chr12:11992106_GTA/GTTA | p.Ala67fs | NA |
| AZA026 | SD | DEC | KDM6A | chrX:44937692_TACA/TA | p.Thr1013fs | NA |
| AZA026 | SD | DEC | RUNX1 | chr21:36259150_ATGGGCAGGGTC/ATGGGCAGGGTTGGGCAGGGTC | p.Ile114fs | NA |
| AZA026 | SD | DEC | SRSF2 | Chr17: 74732959 | p.Pro95Leu | COSM146288 |
| AZA026 | SD | DEC | STAG2 | chrX:123191791_TGT/TGGT | p.Val461fs | NA |
| AZA027 | SD v HI | DEC | RAD21 | chr8:117864932_GACA/GACG | p.Cys392Arg | NA |
| AZA027 | SD w HI | INC | DNMT3A | chr2:25459836_T/C | p.Gln816Arg | NA |
| AZA027 | SD w HI | ST | SMC1A | chrX:53442031_T/C | p.His66Arg | NA |
| AZA027 | SD w HI | ST | TET2 | chr4:106157510_TGG/TG | p.Gly805fs | NA |
| AZA028 | CR short | NA | NA | NA | NA | NA |
| AZA029 | CR short | NA | NA | NA | NA | NA |
| AZA030 | SD w HI | INC-DEC | ATRX | chrX: 76856021 | p.Asn1860Ser | COSM5001991 |
| AZA030 | SD w HI | INC-DEC | EZH2 | hr7: 148525904 | p.Asp185His | COSM3762469 |
| AZA030 | SD w HI | INC-DEC | TP53 | chr17: 7577574 | p.Tyr236Cys | COSM116672 |
| AZA030 | SD w HI | ST | CUX1 | chr7:101844830_GCCCA/GCCCC | p.Thr764Pro | NA |
| AZA031 | SD w HI | INC | BCOR | chrX:39933839_CGA/CGG | p.Val253Ala | NA |
| AZA031 | SD w HI | INC | HRAS | chr11:533886_T/C | p.Asp57Gly | NA |
| AZA031 | SD w HI | INC | IDH2 | chr15: 90631835 | p.His173Pro | COSM3999681 |
| AZA031 | SD w HI | INC | ZRSR2 | chrX:15808619_AT/AA | start loss | NA |
| AZA032 | PG | ST | ASXL1 | chr20:31023472 | p.Asn986Ser | COSM96383 |
| AZA032 | PG | ST | EZH2 | chr7: 148525904 | p.Asp185His | COSM3762469 |
| AZA032 | PG | ST | TP53 | chr17:7577505 | p.Asp259Val | COSM1646864 |
| AZA033 | PG | INC-DEC | BCOR | chrX: 39914723 | p.Arg1547* | COSM39412 |
| AZA033 | PG | ST | BCORL1 | chr19:33792348 | p.Asp355fs | NA |
| AZA033 | PG | ST | CEBPA | chrX: 129147373 | p.Gly209Ser | COSM5019165 |
| AZA034 | PG | DEC | CUX1 | chr7:101844790_TCACCATCCTCA/TCCCCACCCCCC | p.ThrIleLeuThr750ProThrProPro | NA |
| AZA034 | PG | ST | BCORL1 | chrX: 129147373 | p.Gly209Ser | COSM5019165 |
| AZA034 | PG | ST | CDKN2A | chr9: 21971137 | p.Asp74Ala | COSM4163710 |
| AZA034 | PG | ST | TP53 | chr17:7572977_AGGTAGAC/GGGGAGAC | p.ThrSer377ProPro | NA |
| AZA034 | PG | ST | TP53 | chr17:7572977_AGGTAGAC/GGGGAGAC | p.ThrSer377ProPro | NA |
| AZA035 | PG | DEC | TET2 | chr9:139396754_T/C | p.Glu1785Gly | NA |
| AZA035 | PG | INC | CSFR3 | chr1:36932875_C/T | p.Ala666Thr | NA |
| AZA035 | PG | INC | CUX1 | chr7:101882599_GCCT/GCCC | p.Tyr1220His | NA |
| AZA035 | PG | ST | BCORL1 | chrX: 129147373 | p.Gly209Ser | COSM5019165 |
| AZA037 | SD v HI | DEC | GATA2 | chr3:128200133_T/C | p.Glu391Gly | NA |
| AZA037 | SD w HI | INC | BCOR | chrX:39911567_T/C | p.Glu1688Gly | NA |
| AZA038 | SD w HI | INC | PHF6 | chrX:133551261_GACTT/GT | p.Thr300del | NA |
| AZA038 | SD w HI | INC | RUNX1 | chr21: 36252878 | p.Arg162Gly | COSM24718 |
| AZA038 | SD w HI | INC-DEC | RUNX1 | chr21:36164455_C/A | p.Glu474* | NA |
| AZA038 | SD w HI | INC-DEC | RUNX1 | chr21:36171760_C/T |  | NA |
| AZA038 | SD w HI | ST | SRSF2 | chr17: 74732959 | p.Pro95His | COSM211504 |
| AZA039 | SD | INC | ASXL1 | chr20: 31022238 | p.Gln575* | COSM133034 |
| AZA039 | SD | INC | SF3B1 | chr2: 198266834 | p.Lys700Glu | COSM84677 |
